# Antipsychotic polypharmacy prescribing and risk of hospital readmission

**DOI:** 10.1007/s00213-017-4767-6

**Published:** 2017-10-28

**Authors:** Giouliana Kadra, Robert Stewart, Hitesh Shetty, James H. MacCabe, Chin-Kuo Chang, Jad Kesserwani, David Taylor, Richard D. Hayes

**Affiliations:** 1 Institute of Psychiatry, Psychology and Neuroscience, King’s College London, BRC Neucleus, Mapother House, De Crespigny Park, London, SE5 8AF UK; 2 South London and Maudsley NHS Foundation Trust, London, UK

**Keywords:** Co-prescribing, Polypharmacy, Antipsychotics, Rehospitalisation, Readmission

## Abstract

**Objectives:**

The aim of this study was to determine if there was an association between being discharged on antipsychotic polypharmacy (APP) and risk of readmission into secondary mental health care.

**Methods:**

Using data from the South London and Maudsley (SLAM) case register, service users with serious mental illness (SMI), discharged between 1st January 2007 and 31th December 2014, were followed up for 6 months. Patients were classified as receiving either monotherapy or polypharmacy at index discharge. Multivariable Cox regression models were constructed, adjusting for sociodemographic, socioeconomic, clinical and service use factors.

**Results:**

We identified 5523 adults who had been admitted at least once to SLAM, of whom 1355 (24.5%) were readmitted into secondary mental health care. In total, 15% (*n* = 826) of patients were discharged on APP and 85% (*n* = 4697) on monotherapy. Of these, 30.9% (*n* = 255) and 23.4% (*n* = 1100) were readmitted respectively. Being discharged on APP was associated with a significantly increased risk of readmission, in comparison to patients discharged on monotherapy (HR = 1.4, 1.2–1.7, *p* < 0.001). This association was maintained in the fully adjusted model and following several sensitivity analyses. We further established that patients receiving clozapine APP (*n* = 200) were at a significantly increased risk for readmission in comparison to patients on clozapine monotherapy (HR = 1.8, 1.2–2.6, *p* = 0.008).

**Conclusions:**

Our results suggest that patients discharged on APP are more likely to be readmitted into hospital within 6 months in comparison to those discharged on monotherapy. This needs to be considered in treatment decisions and the reasons for the association clarified.

## Introduction

An additional regular antipsychotic is frequently added to treatment [as opposed to pro re nata (PRN)] in inpatient settings to manage residual clinical symptoms following monotherapy (Centorrino et al. 2008; Grech and Taylor 2012). However, antipsychotic polypharmacy (APP) has not been found to be associated with more clinical improvement from the time of admission, to the point of discharge, in comparison to monotherapy (Centorrino et al. 2005; Centorrino et al. 2004; Biancosino et al. 2005), and little is currently known about APP, once patients return to the community.

Hospital readmission rates are high amongst individuals with serious mental illness (SMI) (Schennach et al. 2012), with the risk for rehospitalisation peaking in the first months after discharge (Bodén et al. 2011). Factors that have been associated with an increased risk for readmission are shorter hospital stays (Boaz et al. 2013), medication non-adherence (Haddad et al. 2014) and comorbid substance use (Boaz et al. 2013).

Research examining predictors of APP has indicated that patients with higher inpatient and outpatient contact (Kadra et al. 2016; Ortiz et al. 2016; Centorrino et al. 2004; Kreyenbuhl et al. 2007; Connolly and Taylor 2014) and greater illness severity (Kadra et al. 2016; Correll and Gallego 2012) are at particular risk of receiving APP prescription. However, research examining how patients on APP fare post discharge has been sparse (Correll et al. 2009) and contradictory. For example, evidence has been mainly derived from medical health insurance records, with findings indicating that the choice between APP or monotherapy has no effect on the risk for readmission (Boaz et al. 2013); and that APP is associated with lower hospital readmission in comparison to monotherapy (Katona et al. 2014). There has been sparse evidence to suggest that clozapine is associated with reduced rehospitalisation (Nielsen et al. 2012; Gee et al. 2016; Tiihonen et al. 2017), and clozapine augmentation is currently the only APP regime that has some empirical support (Freudenreich and Goff 2002; Taylor et al. 2011), hence its acceptance as a third-line treatment for SMI (NCCMH 2010). However, it is unclear whether people receiving clozapine polypharmacy differ in the risk of readmission. Therefore, the aim of this study was to determine if there was an association between being discharged on APP and risk of readmission, once patients return to the community, using a large cohort of de-identified electronic health records. Furthermore, we set out to investigate if the inclusion of clozapine in APP had an impact on this risk.

## Methods

We carried out an observational cohort study using anonymised data from South London and Maudsley (SLAM) NHS Foundation Trust electronic health records (EHRs), collected retrospectively for the time period between 1st January 2007 and 31st December 2014. SLAM is one of the largest providers of secondary health care in Europe, serving four London boroughs (Lambeth, Southwark, Lewisham and Croydon) and a catchment population of approximately 1.36 million (Stewart et al. 2009; Perera et al. 2016). The Clinical Record Interactive Search (CRIS) system was developed in 2008 to allow researchers to search and retrieve anonymised SLAM EHRs within a robust governance framework. Currently, over 280,000 cases are represented. CRIS was approved by the Oxfordshire Research Ethics Committee C (reference 08/H606/71+5) in 2008.

### Selection criteria and primary outcome

We identified all adults who had received a SMI diagnosis such as schizophrenia (ICD-10 code: F20.x), schizoaffective disorder (F25.x) or bipolar disorder (F31.x) between 1st January 2007 and 31st December 2014. A decision to include all three of the above diagnoses was made based on discussions with clinicians within the service and previous published literature. More specifically, clinical symptoms are believed to lie on a continuum between these diagnoses and it is not uncommon that a diagnosis is changed over the course of the patient’s illness (Esterberg and Compton 2009). Furthermore, previous research (Grech and Taylor 2012) has indicated that a proportion of patients prescribed long-term antipsychotic polypharmacy have a bipolar affective disorder diagnosis. We further identified all patients with at least one inpatient admission during the observation period and who were residents in the boroughs of SLAM. Patients residing outside the local catchment area can be referred to SLAM services for specialist treatment, due to particularly severe or treatment-resistant symptoms. However, these patients return to their borough of residence following discharge, and therefore follow-up for readmission is not possible for this group. Therefore, this group was excluded. For patients with multiple admissions, we selected admissions that were followed by a discharge on clozapine either as a single antipsychotic or as part of polypharmacy; otherwise, the first recorded admission was used. This was based on previous evidence suggesting that clozapine is often under-prescribed in relation to other antipsychotics and to polypharmacy (Lochmann van Bennekom et al. 2013), so we sought to identify as many cases as we could to increase statistical power sufficient to carry out an analysis for this group. We followed up all patients from the point of their index inpatient discharge for a 6-month period to establish whether or not they were readmitted into secondary mental health care. Previous research indicates that the risk for readmission is highest in the 30 days post inpatient discharge (Boaz et al. 2013), and we reasoned that a 6-month window would thus capture most readmissions. Readmissions data were derived from structured fields in CRIS. Follow-up stopped at the first hospital readmission, date of death, or 31st December 2014, whichever occurred first. Date of death within the observation window was traced for the entire cohort through routine nationwide mortality tracing linked to the electronic health record and carried out on a monthly basis (Perera et al. 2016).

### Data extraction

We extracted clinical information in the EHR through CRIS from structured and unstructured fields (free-text fields such as clinician–patient encounters and correspondence between health care professionals). For antipsychotic prescribing, we also used information available from SLAM pharmacy records. We examined all antipsychotic drugs listed in the British National Formulary (BNF) 65. Antipsychotic medication data in free text was also extracted using a natural language processing (NLP) information extraction application developed using General Architecture for Text Engineering (GATE) software (Cunningham et al. 2013), a suite of tools that facilitates the use and development of NLP applications and features, and which has been used to derive a large volume of meta-data in CRIS for previous and current research (Perera et al. 2016; Kadra et al. 2015). NLP applications take into account the linguistic context when extracting data from free text, therefore offering a more sophisticated approach of extracting information than basic key word searches.

### Exposures of interest and other covariates

We examined individual EHRs to ascertain whether patients were discharged on a single antipsychotic (i.e. monotherapy) or two or more antipsychotics (i.e. APP). Antipsychotic regimen was determined by a patient being prescribed the same antipsychotic/s during their inpatient stay and in the 6 weeks following their discharge. In addition, we extracted a number of sociodemographic, socioeconomic, clinical and service use features.

Age, gender, ethnicity and relationship status were derived from structured fields in CRIS. Age was calculated at index discharge. A likelihood ratio test indicated that it was appropriate to use age as a continuous variable in the analysis. Seventeen ethnic groups were collapsed into six categories (“British”, “Other White”, “Asian”, “Caribbean”, “African” and “Other”) due to small numbers in some cells. Relationship status was defined as “relationship” (cohabitating, married or civil partnership) and “no relationship” (single, divorced, separated, widowed, unknown). We used an area-level index of multiple deprivation to estimate socioeconomic status based on seven domains of deprivation ascertained from 2007 UK Census estimates (employment, income, education, health, barriers to housing and services, crime and living environment), which are weighted and combined into an overall score applied to a given geographic area (DCLG 2011). In this case, multiple deprivation indices were applied to lower super output areas (LSOAs), each containing on average 1500 residents (DCLG 2011). LSOAs were categorised in tertiles in the analysis.

Clinical symptoms were evaluated through Health of the Nation Outcome Scales (HoNOSs) completed in routine clinical practice, prioritising those completed on or before the index discharge date. In cases where a HoNOS at or prior to discharge was not available, we took the closest score available after the discharge date. HoNOS is a clinical outcome instrument in wide routine use, composed of 12 items designed to measure behaviour, impairment, symptoms and social functioning (Wing et al. 1998). Items are scored on a scale of 0 (no problem) to 4 (severe to very severe problem). Due to small cell sizes, subscale scores were collapsed into three categories: 0 “not a problem”; 1 “minor problem requiring no action”; 2–4 “significant problem”. We further ascertained whether or not the patient had received a mental illness diagnosis due to alcohol (ICD 10: F10) or opioid use (ICD 10: F11) prior to the index discharge. This information was extracted from structured and free-text fields. We extracted two measures of prior service use: (1) the number of days spent as an inpatient in the 6 months prior to the index discharge date; and (2) the proportion of face-to-face contact received as an outpatient in the 6 months prior to the index discharge (multiple events on a single day were counted as 1 day of clinical contact, whilst clinical contact with outpatient services during an inpatient admission was not counted). Both variables were categorised in tertiles. For the purpose of a sensitivity analysis, we tried to establish medication non-adherence, by ascertaining whether patients had ever been previously on a community treatment order (CTO) [CTO refers to a conditional discharge from inpatient admission, commonly implemented for a period of 6 months to improve adherence to medication and promote regular contact with services (DoH 2007)] and antipsychotic long-acting injectable (LAI) prescription. This information was derived from structured and free-text fields and categorised as a binary variable, 0 (no previous history of CTO and LAI use) and 1 (previous history of both CTO and LAI use).

### Statistical analysis

STATA 13 was used to conduct all statistical analyses. Sample characteristics were summarised by percentage of readmission for the total cohort and by antipsychotic group. Kaplan–Meier curves with a log-rank test were used to compare those who were prescribed APP and monotherapy in relation to readmission. Following checks of proportional hazard assumptions, Cox regression procedures were used to examine the associations between APP and risk of readmission.

Possible confounders were decided on a priori, based on their plausibility as potential confounders and evidence from previous literature. Age, gender, ethnicity, relationship status, deprivation status, clinical symptoms (HoNOSs), comorbid diagnoses and service use in the 6 months prior to the index discharge date were included as covariates in the multivariable analysis. We further conducted several sensitivity analyses to test whether any possible associations between APP and hospital readmissions were maintained after removing factors that may have had an effect: (1) we excluded patients with prior history of CTO and LAI use. The above are potential markers of non-adherence and therefore important to account for when considering medication use. (2) We restricted the analysis to all patients with a diagnosis of schizophrenia (ICD 10: F20) in order to test if the association was maintained for this group. (3) We excluded patients from the borough of Lewisham as they did not have SLAM pharmacy data (however, they did have medication data from structured and free-text fields in PJS). (4) We excluded patients with HoNOS score obtained after the index discharge. (5) We restricted the analysis to patients who had not been prescribed clozapine. (6) To reduce the effect of confounding by indication, we used a standard propensity score method, where the propensity score was the probability of being placed on polypharmacy at index discharge where all the potential confounders described in Table 1 were included in the model. The propensity scores were then used as a covariate in place of all of the aforementioned confounders (i.e. sociodemographic, socioeconomic, clinical and service use) in the Cox model. Propensity score was further used to identify patients who were at risk of being prescribed monotherapy and polypharmacy at discharge. We then constructed a fully adjusted Cox model and restricted the analysis to patients with this restricted range of propensity scores. Finally, we carried out a fully adjusted Cox model, where patients on clozapine APP and non-clozapine polypharmacy were compared to patients on clozapine monotherapy on their risk of hospital readmission. In this latter analysis, clozapine monotherapy was considered to be clinically the most meaningful reference group. Clozapine prescribing often involves a period of clinical discussion, as well as physical and blood checks. Therefore, patients who are initiated on clozapine could be somewhat different to patients that have not been initiated on clozapine. Therefore, restricting this latter analysis to patients that have been prescribed clozapine also reduces confounding by indication.

**Table 1:** Sample characteristics by antipsychotic regimen prescribed at index discharge^a^ (*N* = 5523)

Variables	Total sample *n*	Antipsychotic monotherapy *n* (%)	Antipsychotic polypharmacy *n* (%)
Sociodemographic and socioeconomic factors			
Age			
Mean (SD)	41.3 (14.5)	41.3 (14.7)	41.4 (13.1)
Gender			
Female	2573	2185 (46.5)	388 (47.0)
Male	2950	2512 (53.5)	438 (53.0)
Ethnicity group^a^			
British	1662	1447 (30.8)	215 (26.0)
Other White	453	383 (8.2)	70 (8.5)
Asian	334	285 (6.1)	49 (5.9)
Caribbean	730	596 (12.7)	134 (16.2)
African	1926	1623 (34.6)	303 (36.7)
Other	418	363 (7.7)	55 (6.7)
Relationship status			
No relationship	4806	4083 (86.9)	723 (87.5)
Relationship	717	614 (13.1)	103 (12.5)
Deprivation level in area of residence			
Low level	1834	1548 (33.0)	286 (34.6)
Medium level	1844	1581 (33.7)	263 (31.8)
High level	1845	1568 (33.4)	277 (34.0)
Clinical factors			
Diagnosis^a^			
Schizophrenia (ICD-10: F20)	3706	3103 (66.1)	603 (73.0)
Schizoaffective disorder (ICD-10: F25)	490	386 (8.2)	104 (12.6)
Bipolar affective disorder (ICD-10:F31)	1327	1208 (25.7)	119 (14.4)
Overactive and aggressive behaviour			
Not a problem	3081	2625 (56.4)	456 (55.8)
Minor problem	1222	1039 (22.3)	183 (22.4)
Significant problem	1166	987 (21.2)	179 (21.9)
Depressed mood			
Not a problem	2769	2335 (50.3)	434 (53.2)
Minor problem	1574	1341 (29.0)	233 (28.6)
Significant problem	1119	970 (20.9)	149 (18.3)
Non-accidental self-injury			
Not a problem	4829	4105 (88.3)	724 (88.5)
Minor problem	312	257 (5.5)	55 (6.7)
Significant problem	326	287 (6.2)	39 (4.8)
Physical illness or disability			
Not a problem	3715	3177 (68.5)	538 (65.9)
Minor problem	824	689 (14.9)	135 (16.5)
Significant problem	917	774 (16.7)	143 (17.5)
Hallucinations and delusions^a^			
Not a problem	1824	1609 (34.7)	215 (26.3)
Minor problem	1208	1023 (22.1)	185 (22.7)
Significant problem	2423	2008 (43.3)	415 (51.0)
Problems with activities of daily living			
Not a problem	2791	2405 (52.1)	386 (47.7)
Minor problem	1376	1150 (24.9)	226 (27.9)
Significant problem	1256	1059 (23.0)	197 (24.4)
Problems with living conditions			
Not a problem	3069	2559 (57.6)	470 (59.7)
Minor problem	1126	974 (21.6)	152 (19.3)
Significant problem	1106	941 (20.8)	165 (21.0)
Problems with occupation			
Not a problem	2179	1865 (41.3)	314 (39.8)
Minor problem	1542	1302 (28.9)	240 (30.4)
Significant problem	1580	1344 (29.8)	236 (29.9)
Problems with relationships			
Not a problem	2199	1883 (40.9)	316 (39.1)
Minor problem	1590	1343 (29.1)	247 (30.5)
Significant problem	1628	1382 (30.0)	246 (30.4)
Prior alcohol use (ICD-10:F10)			
No	5053	4300 (91.5)	753 (91.2)
Yes	470	397 (8.5)	73 (8.8)
Prior opioid use (ICD-10:F11)			
No	5442	4624 (98.4)	818 (99.0)
Yes	81	73 (1.6)	8 (1.0)
Service use			
Days of inpatients stay in the previous 6 months (tertiles)^a^			
0–24 days	1777	1573 (34.5)	204 (24.7)
25–65 days	1904	1643 (35.0)	261 (31.6)
66–185 days	1842	1481 (31.5)	361 (43.7)
Days of outpatient contact in the previous 6 months (tertiles)^a^			
1–2 days	1112	979 (28.1)	133 (22.0)
3–8 days	1502	1294 (37.1)	208 (34.4)
9–117 days	1479	1215 (34.8)	264 (43.6)

^a^There is a statistically significant difference (*p* < 0.05) between antipsychotic monotherapy and polypharmacy

## Results

In total, 5523 individuals met the inclusion criteria for the study. Table 1 describes the characteristics of the total cohort and by antipsychotic regimen. Antipsychotic monotherapy and polypharmacy were very similar in their sociodemographic and socioeconomic composition. However, there was a higher proportion of British patients in the monotherapy group, whereas the polypharmacy group had a higher proportion of patients from black African and black Caribbean ethnic backgrounds. Furthermore, patients on antipsychotic monotherapy were more likely to have been diagnosed with bipolar affective disorder (ICD 10: F31), whereas patients prescribed APP were more likely to receive schizophrenia diagnosis. Patients discharged on APP were also more likely to have significant problems with hallucinations and/or delusions, and had more contact with services in the previous 6 months (both inpatient and outpatient).

Table 2 summarises readmission across the antipsychotic regimens. Twenty-five per cent (*n* = 1355) of the sample were readmitted within 6 months post discharge. In total, 15% (*n* = 826) of patients were discharged on APP and 85% (*n* = 4697) patients were discharged on monotherapy. Of these, 30.9% (*n* = 255) and 23.4% (*n* = 1100) were readmitted respectively.

**Table 2:** Hospital readmission by antipsychotic regimen

Variables	Total *N*	Readmitted *n* (%)	Not readmitted *n* (%)
Total	5523	1355 (24.5)	4168 (75.5)
Monotherapy	4697	1100 (23.4)	3597 (76.6)
Clozapine monotherapy	395	85 (21.5)	310 (78.5)
Antipsychotic polypharmacy	826	255 (30.9)	571 (69.1)
Clozapine polypharmacy	200	63 (31.5)	137 (68.5)

**Fig. 1:**
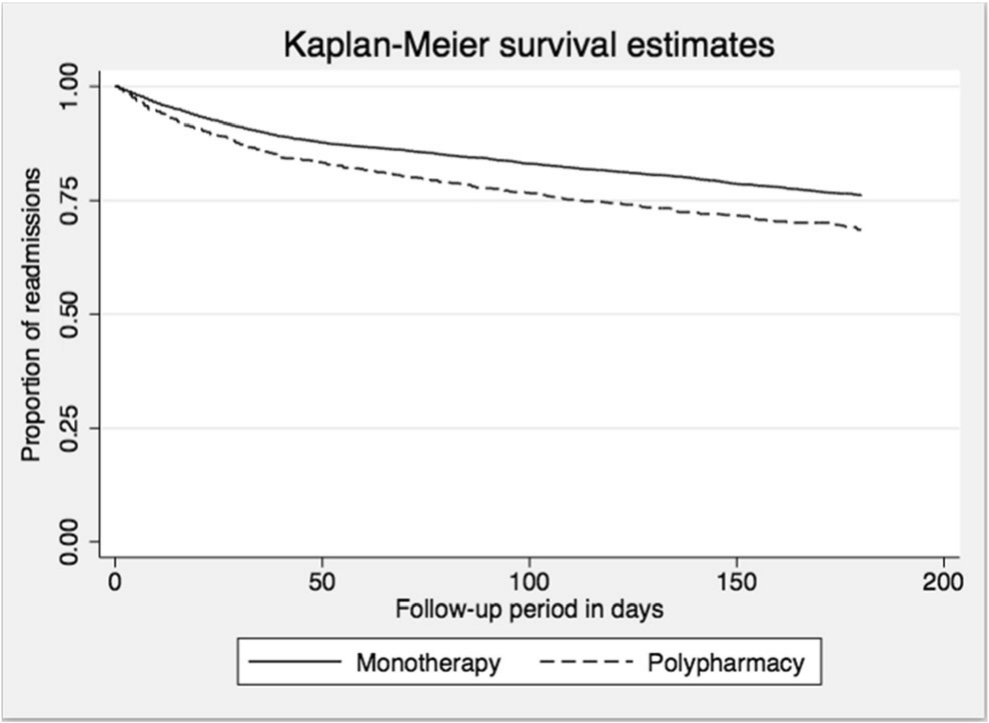
Kaplan–Meier survival curves displaying the readmission status of people with serious mental illnesses comparing those discharged on antipsychotic monotherapy to those discharged on polypharmacy (*n* = 5523) (*p* < 0.001)

Figure 1 presents the Kaplan–Meier curves comparing readmission over time for patients discharged on either antipsychotic monotherapy or polypharmacy. Those prescribed monotherapy displayed significantly less readmission (*p* < 0.001) over time.

Table 3 summarises Cox proportional hazards models for the association between being discharged on APP and secondary mental health care readmission. In summary, APP was associated with a significantly increased risk for hospital readmission; this association was sustained after adjusting for a number of sociodemographic, socioeconomic, clinical and service use factors, and changed little after alternative adjustment for propensity scores. We further conducted a number of sensitivity analyses, also described in Table 3, which again had little impact on the main outcome.

**Table 3:** Multivariable Cox regression analysis of the association between antipsychotic polypharmacy (APP) prescribing and hospital readmission in individuals with serious mental illness

Regression model	Association between APP^a^ and hospital readmission	
	HR (95% CI)	*p* value
Unadjusted model	1.4 (1.2–1.6)	*p* < 0.001
Model adjusted for sociodemographic and socioeconomic factors	1.4 (1.2–1.6)	< 0.001
Model adjusted for clinical symptoms	1.4 (1.3–1.7)	*p* < 0.001
Model adjusted for service use in the previous 6 months	1.3 (1.1–1.6)	*p* < 0.001
Model adjusted for all of the above factors	1.4 (1.2–1.7)	*p* < 0.001
Alternative model adjusted for propensity score as a covariate	1.4 (1.2–1.7)	*p* < 0.001
Sensitivity analyses		
Analysis excluding patients on community treatment orders (CTOs) and previously prescribed long-acting injectables (LAIs)	1.3 (1.1–1.6)	*p* = 0.006
Analysis restricted to patients with schizophrenia diagnosis (ICD 10: F20)	1.5 (1.2–1.8)	*p* < 0.001
Analysis excluding patients from the borough of Lewisham	1.4 (1.2–1.8)	*p* < 0.001
Analysis excluding patients who have obtained their HoNOS score after the index antipsychotic prescription	1.4 (1.2–1.7)	*p* < 0.001
Analysis restricted to patients who were at risk of being prescribed both monotherapy and polypharmacy (based on propensity scores)	1.4 (1.2–1.7)	*p* < 0.001
Analysis restricted to patients without clozapine	1.4 (1.1–1.6)	*p* = 0.001

*n* = 5523 individuals, 1355 readmissions

^a^Monotherapy is used as the reference group

Clozapine polypharmacy constituted 4% of the sample (*n* = 200), whereas non-clozapine polypharmacy was 11.3% (*n* = 626). A fully adjusted Cox proportional hazards model indicated that clozapine APP was associated with significantly increased risk for readmission in comparison to clozapine monotherapy (HR = 1.8, 1.2–2.6, *p* = 0.008) (Table 4). However, when we compared the risk for readmission between clozapine monotherapy and non-clozapine APP, we found no significant difference between the two regimens (HR = 1.4, 0.9–1.9, *p* = 0.063).

**Table 4:** Multivariable Cox regression analysis of the association between clozapine and non-clozapine antipsychotic polypharmacy prescribing and hospital readmission in individuals with serious mental illness

	Clozapine polypharmacy (*n* = 200)		Non-clozapine polypharmacy (*n* = 626)	
Models^a^	HR (95% CI)	*p* value	HR (95% CI)	*p* value
Unadjusted model	1.6 (1.2–2.2)	*p* = 0.004	1.6 (1.2–2.0)	*p* < 0.001
Model adjusted for sociodemographic and socioeconomic factors	1.6 (1.2–2.3)	*p* = 0.003	1.7 (1.3–2.2)	*p* < 0.001
Model adjusted for clinical symptoms	1.7 (1.2–2.4)	*p* = 0.003	1.5 (1.1–1.9)	*p* = 0.004
Model adjusted for service use in previous 6 months	1.6 (1.1–2.4)	*p* = 0.012	1.4 (1.0–1.9)	*p* = 0.031
Fully^b^ adjusted model	1.8 (1.2–2.6)	*p* = 0.008	1.4 (0.9–1.9)	*p* = 0.063

*n* = 1221; readmissions = 340

^a^Clozapine monotherapy group has been used as the reference

^b^Adjusted for all sociodemographic, socioeconomic, clinical and service use factors described in Table 1

## Discussion

This study examined the association between being discharged on APP from inpatient settings and subsequent mental health care readmissions, in a retrospective analysis of a large cohort of patients, taking into account a wide range of other covariates. In summary, we found that patients discharged on APP (whether including clozapine or not) were at an increased risk of rehospitalisation. This association remained statistically significant and relatively unaltered in strength after multiple adjustments, sensitivity analyses and the use of propensity score methods to address confounding by indication. The results further indicated that patients discharged on clozapine polypharmacy had an even greater risk for readmission when compared to patients on clozapine monotherapy.

Previous research on APP as a predictor of readmission has been sparse and inconclusive. Our findings were consistent with evidence from clinical record studies (Kreyenbuhl et al. 2007), indicating that patients prescribed APP were more likely to be admitted to secondary mental health care inpatient settings. For example, Kreyenbuhl et al. (2007) found that patients who had an additional antipsychotic prescribed, as opposed to being switched to a different antipsychotic, were three times more likely to be hospitalised. However, our findings were not in agreement with previous research investigating medical insurance records and rehospitalisation amongst patients prescribed long-term APP (Boaz et al. 2013; Katona et al. 2014). For example, Boaz and colleagues (Boaz et al. 2013) found that polypharmacy at discharge was not associated with future hospital readmissions; rather, readmission was associated with patients being insufficiently stable at the point of initial discharge. Greater clinical severity in patients prescribed APP is one possible mechanism proposed to explain the higher level of readmission, and is consistent with the associations we found for APP at discharge with schizophrenia diagnosis, positive symptoms and higher service contact (Kadra et al. 2016; Correll and Gallego 2012). However, the association with readmission persisted and was largely unaltered after adjusting for these factors. Adjusting for other factors known to affect levels of readmission such as possible medication non-adherence (Haddad et al. 2014) as indicated by previous CTOs and LAI prescription, and substance use (Boaz et al. 2013), also made little difference to the results. Furthermore, the association between polypharmacy and readmission was sustained after restricting the analysis to patients who potentially might have been prescribed antipsychotic monotherapy or polypharmacy based on their propensity scores. We found no evidence to suggest that APP (whether this was clozapine or non-clozapine) was associated with a lower risk for readmission, as indicated by Katona et al. (2014). An important caveat to consider is that despite general consensus across countries with regard to treatment guidelines (APA 2010; NICE and NCCMH 2013), it is possible that clinical practices do differ, and the aforementioned evidence reflects true differences in prescribing across countries.

The results further indicated that this risk was significantly higher for patients prescribed clozapine polypharmacy as compared to clozapine monotherapy. The same pattern was not observed for patients on non-clozapine polypharmacy. Existing research, mainly based on randomised controlled trials and open-label trials, examining clozapine polypharmacy has indicated little to no benefit of this regimen in improving residual clinical symptoms (Freudenreich and Goff 2002; Taylor et al. 2011), and our results supported this, by confirming that clozapine polypharmacy does not appear to reduce the risk for readmission for patients with SMI. In addition, our findings further suggested that within the groups of patients receiving APP, there could be a sub-population that is at a particularly high risk for readmission. This could be due to a number of different factors (such as severity of clinical symptoms), which need further investigation.

This study had several strengths. SLAM, in common with other UK secondary mental health services, is a near-monopoly provider for its geographic catchment (Perera et al. 2016), increasing the potential generalisability of findings and maximising their reflection of real-world clinical practice (Stewart et al. 2009). In addition, the large cohort provided statistical power to detect the primary association of interest and to adjust for a broad range of potential confounders. All exposures were measured on or before the index discharge, therefore enabling us to make temporal inference with regards to APP and readmission.

There were several potential limitations in this study, which need to be borne in mind. Despite multiple adjustment, residual confounding cannot be excluded absolutely in an observational design. Specifically, we did not capture factors such as time known to services or duration of prior hospital admissions (Boaz et al. 2013). In addition, we were unable to identify the concomitant prescription of other non-antipsychotic drugs, which could have possibly had an effect on readmission. Furthermore, symptom assessment in this study was limited to individual HoNOS items, measured at one point in time. This scale has received some previous criticism with regards to its measurement of symptoms (Bebbington et al. 1999), and we were only able to analyse a composite measure of clinical symptoms and daily functioning. Although we employed propensity score adjustment and restriction, confounding by indication cannot be completely ruled out.

Our findings have several important potential implications. Our results indicated that patients on APP are generally more unwell; therefore, the prescription of regimens that lack empirical support is likely to further increase patient burden already present in this population (Ganguly et al. 2004; Paton et al. 2008). We found that patients receiving clozapine polypharmacy had a particularly elevated risk for readmission as compared to clozapine monotherapy. This is suggestive of potential difference in treatment needs across patients receiving APP, further indicating that this is not a homogenous population. Therefore, future research would benefit from further examining this sub-group in relation to their clinical symptoms, treatment needs and course of antipsychotic medication prescribing (i.e. time from non-clozapine monotherapy to clozapine augmentation). Lastly, the findings provide further support for the need to reduce APP prescribing. APP prescribing has remained widespread not only across clinical services but also across countries and time (Gallego et al. 2012), with a trend that has been resistant to change (Paton et al. 2008) and with a high cost to service. More specifically, APP has been associated with a higher bed occupancy and length of inpatient stay, in addition to extra cost associated with multiple medication prescribing (Baandrup et al. 2012; Gilmer et al. 2007). Evidence from a recent quality improvement programme has indicated that polypharmacy can be reduced successfully (Mace and Taylor 2015). Therefore, there is a clear need for similar programmes to be implemented on a wider national level.
